# MALDI-TOF MS Affords Discrimination of *Deinococcus aquaticus* Isolates Obtained From Diverse Biofilm Habitats

**DOI:** 10.3389/fmicb.2018.02442

**Published:** 2018-10-15

**Authors:** James M. Tuohy, Sabrina R. Mueller-Spitz, Chad M. Albert, Stacy E. Scholz-Ng, Melinda E. Wall, George T. Noutsios, Anthony J. Gutierrez, Todd R. Sandrin

**Affiliations:** ^1^Biology Department, Glendale Community College, Glendale, AZ, United States; ^2^Biology Department, University of Wisconsin Oshkosh, Oshkosh, WI, United States; ^3^Sustainability Institute for Regional Transformations, University of Wisconsin Oshkosh, Oshkosh, WI, United States; ^4^Department of Natural Sciences, Western New Mexico University, Silver City, NM, United States; ^5^School of Mathematical and Natural Sciences, Arizona State University, Glendale, AZ, United States; ^6^Julie Ann Wrigley Global Institute of Sustainability, Arizona State University, Tempe, AZ, United States

**Keywords:** mass spectroscopy, freshwater biofilms, Bruker’s Biotyping, BOX-A1R fingerprinting, 16S rRNA gene

## Abstract

Matrix-assisted Laser Desorption Ionization-Time of Flight Mass Spectroscopy (MALDI-TOF MS) has been used routinely over the past decade in clinical microbiology laboratories to rapidly characterize diverse microorganisms of medical importance both at the genus and species levels. Currently, there is keen interest in applying MALDI-TOF MS at taxonomic levels beyond species and to characterize environmental isolates. We constructed a model system consisting of 19 isolates of *Deinococcus aquaticus* obtained from biofilm communities indigenous to diverse substrates (concrete, leaf tissue, metal, and wood) in the Fox River – Lake Winnebago system of Wisconsin to: (1) develop rapid sample preparation methods that produce high quality, reproducible MALDI-TOF spectra and (2) compare the performance of MALDI-TOF MS-based profiling to common DNA-based approaches including 16S rRNA sequencing and genomic diversity by BOX-A1R fingerprinting. Our results suggest that MALDI-TOF MS can be used to rapidly and reproducibly characterize environmental isolates of *D. aquaticus* at the subpopulation level. MALDI-TOF MS provided higher taxonomic resolution than either 16S rRNA gene sequence analysis or BOX-A1R fingerprinting. Spectra contained features that appeared to permit characterization of isolates into two co-occurring subpopulations. However, reliable strain-level performance required rigorous and systematic standardization of culture conditions and sample preparation. Our work suggests that MALDI-TOF MS offers promise as a rapid, reproducible, and high-resolution approach to characterize environmental isolates of members of the genus *Deinococcus*. Future work will focus upon application of methods described here to additional members of this ecologically diverse and ubiquitous genus.

## Introduction

Rapid, mass spectrometry (MS)-based technologies, particularly MALDI-TOF, have revolutionized microbial characterization and identification in clinical environments over the past decade (Emami et al., 2012; Sandrin et al., 2013; Singhal et al., 2015). More conventional biochemical and morphology-based methods have been complemented or replaced by MS-based technologies. Platforms including Bruker’s Biotyper MS (Schulthess et al., 2016) and Biomerieux’s VITEK MS (Dubois et al., 2012) have become ubiquitous in many medical, clinical, and diagnostic labs (Lévesque et al., 2015). Successes applying MS-based approaches at the species level are well-documented (Dieckmann et al., 2008; Khot and Fisher, 2013), and several recent studies suggest that the taxonomic resolution of MALDI-TOF based approaches may permit reliable strain-level characterization (Vargha et al., 2006; Sandrin et al., 2013).

The vast majority of MS-based applications to microbial characterization have resided in clinical and medical settings. While applications to microorganisms indigenous to natural environments are far less commonly reported in the literature, promising results have been described with microorganisms from several diverse natural environments including plant surfaces (Shroff et al., 2015), soil (Singhal et al., 2015), and cave speleothem surfaces (Zhang et al., 2014; Zhang et al., 2015; Penny et al., 2016). In the last couple of decades, manifold efforts have been made to comprehend the activities, dynamics, functions and structures of microbial communities in various ecosystems. A number of approaches have been employed to interrogate microbial diversity in these ecosystems ranging from conventional phylogenetic studies to more high-throughput metagenomics-based approaches. MALDI-TOF MS may offer a robust, rapid, low cost, and reliable alternative method to characterize new microbial species of ecological interest. Natural environments often harbor microbial taxa not well-represented in commercially available MS databases (Rahi et al., 2016). Successful application of current MS-based methods will likely require adding these microorganisms to commercially available databases or creation of custom databases. In either case, efforts to optimize methods may be required to obtain the requisite high quality reference spectra to include in relevant databases.

The genus *Deinococcus* (Brooks and Murray, 1981) contains over 65 species based upon validly published names^1^. Many of these species have been reported to have extraordinary tolerance to gamma and UV radiation as well as oxidative stress and prolonged desiccation (Mattimore and Battista, 1996; Battista, 1997; Slade and Radman, 2011). Members of the genus also characteristically possess multi-layered cell walls that contain ornithine in their peptidoglycan and lack teichoic acid (Hanninen et al., 2010). These distinctive features may permit adaptation to a wide variety of habitats. *Deinococci* have been isolated from environments as diverse as desert soils (de Groot et al., 2005; Rainey et al., 2005), radioactive sites (Asker et al., 2009, 2017), dust (Weon et al., 2007; Yang et al., 2009), fish (Shashidhar and Bandekar, 2009), aquifers (Suresh et al., 2004), and fresh water (Im et al., 2008; Kämpfer et al., 2008).

The ecological roles and strategies deployed by *Deinococcus* in each of these environments remains enigmatic. Current approaches to the characterization of different species of *Deinococcus* include assaying basic aspects of their physiology including temperature ranges, salt tolerance, color, carbon assimilation, and fatty acid profile in addition to variation in the 16S rRNA gene identity (Bouraoui et al., 2012). The phylogenetic classification of bacteria based on the 16S rRNA gene sequencing assumes that 16S rRNA genes are vertically inherited and therefore are indigenous to each species; however, it is well known that most bacteria contain multiple copies of 16S rRNA genes and the possibility of nucleotide variation as well as these genes being derived through horizontal gene transfer may distort relationships within and between taxa (Poretsky et al., 2014). Unfortunately, there has been limited work to develop rapid typing methods to aid in classification when attempting to amass a collection of related members from natural environments. This deficiency limits our understanding of the biogeography and overall biodiversity of the group as a whole. Our interest stems in discerning commonalities and differences between *Deinococci* isolated from similar yet distinct environments. Given the limitations of existing approaches and the promising results obtained using MS with other genera indigenous to natural environments (Rahi et al., 2016), we wish to further assess the utility of this technology. *D. aquaticus* is a free-living, aerobic, non-motile, gram negative rod that has been previously isolated from fresh-water (Im et al., 2008). Here, we seek to develop, apply, and assess the performance of rapid MALDI-based fingerprinting using a model collection of *Deinococcus* biofilm isolates.

Specifically, the objectives of this work were to: (1) develop rapid sample preparation methods that produce high quality, reproducible MALDI-TOF spectra and (2) compare the performance of MALDI-TOF MS-based profiling to common DNA-based approaches including 16S rRNA gene identity and genetic diversity by BOX-A1R fingerprinting. Our results suggest that MALDI-based approaches are more rapid than conventional approaches and perform at least as well as more conventional methods at characterizing strains of this environmentally diverse genus. Finally, our results suggest that MALDI-TOF spectra may contain features (i.e., biosignatures) indicative of physiological differences in co-occurring populations.

## Materials and Methods

### Isolation and Culture Conditions

**FIGURE 1:**
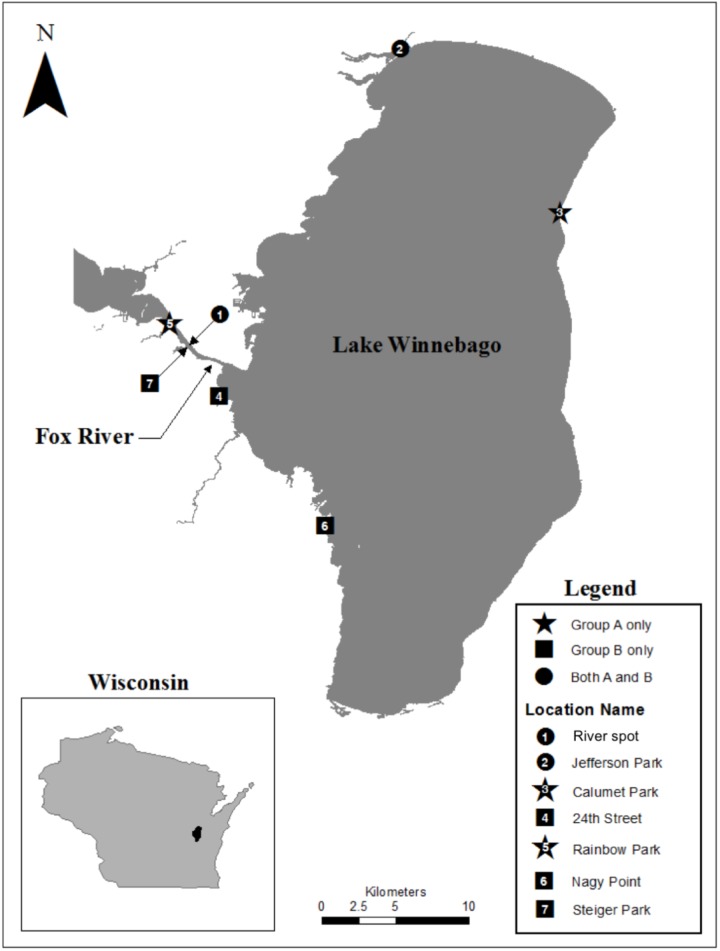
Map of our study area indicating the points where *Deinococcus* isolates were collected within the Fox River – Lake Winnebago ecosystem.

Biofilm samples were collected using environmental sampling swabs with Butterfield’s buffer (Puritan Environmental Sampling Kit, Guilford, ME, United States) from four types of surfaces (e.g., concrete, leaf tissue, metal, and wood). Biofilm samples were collected from surfaces that were entirely submerged in water. The surface was swabbed twice using the same swab following deposition into the Butterfield’s buffer. Sampling occurred between June and July 2013, at seven different locations along the shoreline of Lake Winnebago and the Lower Fox River in Winnebago and Calumet counties, Wisconsin. One isolate, FR100, was cultured from a surface water sample collected from the Fox River on June 2010. Organisms from each swab or water sample were plated in triplicate on R2A agar (Becton Dickinson and Company, Franklin, NJ, United States). The plates were incubated at 20–22°C. The location and isolation habitat for each *Deinococcus* isolate examined are listed in **Table 1**, and a map of the area is depicted in **Figure 1**. Bacterial isolates were preserved in a sterile suspension of 50% glycerol and 50% R2A media at −80°C until further use.

**Table 1:** *Deinococcus* isolates from biofilm communities indigenous to diverse substrates used in the present study.

Isolate	Isolation substrate	Body of water^∗^	Isolation location^#^	GenBank accession^#^
P1	Metal	River	1	MH504166
P2	Metal	River	1	MH504167
P7	Metal	River	1	MH504168
P17	Metal	River	1	MH504169
P21	Metal	Lake	2	MH504170
P22	Metal	Lake	2	MH504171
P23	Metal	Lake	3	MH504172
P34	Wood	Lake	4	MH504173
P41	Concrete	River	5	MH504174
P43	Wood	Lake	6	MH504175
P49^+^	Concrete	River	7	MH504176
P76	Leaf	River	7	MH504184
P65	Wood	Lake	2	MH504185
P71	Concrete	Lake	2	MH504177
P72	Concrete	Lake	2	MH504178
P74	Concrete	Lake	2	MH504179
P79	Concrete	Lake	2	MH504180
P80	Concrete	Lake	2	MH504181
P81	Concrete	Lake	2	MH504182
FR100	Surface Water	River	1	MH504183

*^∗^Fox River or Lake Winnebago; ^#^for isolation location refer to **Figure 1**; ^+^isolate P49 upon analysis turned out to be D. misasensis.*

### Genetic Analysis of Isolates

Pink colonies were selected for isolation and characterization using 16S rRNA gene identity. Genomic DNA was extracted using a PowerLyzer Microbial DNA Isolation kit following the manufacturer’s instructions (Mo Bio Laboratories, Carlsbad, CA, United States). The 16S rRNA gene was amplified using the 8F and 1492R primer set (Lane, 1991; Turner et al., 1999). PCR products were run on a 1% agarose gel in 1× Tris-Acetate-EDTA (TAE) buffer to confirm the appropriate PCR product and then purified using an IBI Gel/PCR DNA Gel Extraction Kit (IBI Scientific, Peosta, IA, United States). The 16S rRNA gene fragments were Sanger sequenced on an 3730 DNA Analyzer (Thermo Fisher Scientific, Waltham, MA, United States), analyzed using Chromas (Technelysium Pty Ltd., South Brisbane, Australia), and assembled using CAP3 (Huang and Madan, 1999). Analysis of the 16S rRNA sequences was performed using MEGA6 (Tamura et al., 2013). Sequences were aligned with Clustal and phylogeny was determined using the neighbor-joining method (Saitou and Nei, 1987).

Intraspecific strain genetic diversity was determined by genomic fingerprinting (Koeuth et al., 1995). Genomic DNA from the 19 isolates was amplified with the BOX-A1R primer, 5′-CTACGGCAAGGC-GACGCTGACG-3′. PCR amplifications were carried out in a 25 μL reaction volume with Phusion High-Fidelity DNA Polymerase (New England Biolabs, Ipswich, MA, United States) supplemented with 3% DMSO following the manufacturer’s protocol (New England Biolabs, Ipswich, MA, United States) and PCR conditions followed those of Koeuth et al. (1995). Three separate PCR reactions were performed for each isolate. PCR products were analyzed on 1.5% agarose gels run in 1X TAE buffer for 5.5 h at 130 V. All gels included three to four lanes of a 0.2–10 kb molecular ladder (New England Biolabs, Ipswich, MA, United States). Gels were post-stained with ethidium bromide and imaged using a Gel Doc XR+ system (Bio-Rad, Hercules, CA, United States). BOX-A1R genomic fingerprints (**Figure 5C**) were analyzed with GelCompar II (Applied Mathematics, Kortrijk, Belgium, version 6.0). Dendrograms were built with the UPGMA algorithm and the Pearson coefficient. Jackknife analysis was performed as described before using average similarities to quantify rates of correct classification to body of water with regards to BOX-A1R fingerprints (Giebel et al., 2008).

### Sample Preparation and Protein Extraction for MALDI-TOF

Protein extraction and sample preparation were performed as previously described (Barbano et al., 2015). Briefly, *D. aquaticus* preserved isolates were plated and streaked for single colonies in Tryptone Glucose Yeast (TGY) Agar plates (Sigma Aldrich, St. Louis, MO, United States). Single colonies from each isolate were cultured in 5 mL of TGY media (Sigma Aldrich) at 20–22°C until cultures reached logarithmic phase, as assessed by measuring optical density at 600 nm to be 1.0 ± 0.01. Bacteria (1 mL) were pelleted by centrifugation at 17,000× *g* for 3 min at RT, washed with sterile ddH_2_O (Millipore, Bedford, MA, United States) and inactivated by re-suspension in 1 mL 75% (v/v) ethanol at RT for 1 h. Verification of inactivation was performed by plating 50 μL of the resuspended bacteria on TGY Agar plates. No CFU were observed after 5 days of incubation at 22°C, demonstrating successful inactivation. Inactivated bacteria were washed with 1 mL ddH_2_O, centrifuged at 10,000× *g* for 3 min at RT and air-dried for 1 min. Bacterial cell walls were disrupted by adding 25 μL of 70% (v/v) formic acid (Millipore Sigma, St. Louis, MO, United States) followed by 25 μL of acetonitrile (Sigma Aldrich). Cells were centrifuged at 17,000× *g* for 3 min at RT and the supernatant containing the protein extract was transferred into a sterile 1.5 mL microcentrifuge tube and stored at −80°C until further use. Bacterial protein extracts (1.0 μL) were pipetted onto a polished steel 96-well MALDI target plate (Bruker Daltonics, Billerica, MA, United States) and air-dried for 15 min. Extracts were spotted onto predetermined, randomly distributed locations on the target plate and were overlaid with 1.0 μL of α-cyano-4-hydroxycinnamic acid (Acros, Fair Lawn, NJ, United States) matrix prepared in 50% acetonitrile and supplemented with 2.5% trifluoroacetic acid (Millipore Sigma, St. Louis, MO, United States). Each isolate was spotted in at least three technical replicates per biological replicate. *D. gobiensis* strain DSM 21396, obtained from Deutsche Sammlung von Mikroorganismen und Zellkulturen (DSMZ, Braunschweig, Germany), was processed, cultured in a similar fashion, and served as the control of our study.

### MALDI-TOF MS Data Acquisition and Data Analysis

Matrix-assisted Laser Desorption Ionization-Time of Flight Mass Spectroscopy data were obtained using a nitrogen laser (λ = 337 nm)-equipped Bruker’s Microflex LRF MALDI-TOF mass spectrometer (Bruker Daltonics, Billerica, MA, United States) under the control of FlexControl software (version 3.0; Bruker Daltonics). Each sample’s spectrum was obtained in a linear, positive ion mode. The spectrometer was calibrated externally using ACTH (1–17) (2094.427 Da), ACTH (18–39) (2466.681 Da), insulin oxidized B (3494.651 Da), insulin (5734.518 Da), cytochrome C (12360.974 Da), and myoglobin (16952.306 Da) prior to each run. Data acquisition was performed automatically in steps of 100 shots for a total of 500 shots. Laser power was set to the necessary minimum power for ionization of selected samples before starting the analyses. The signal-to-noise threshold was set at two, the minimum intensity threshold at 100, and the maximum number of peaks to 500. Peak width was set at 10 m/z and a height of 80%.

Mass spectra were exported from FlexAnalysis as text files (.txt) and imported into BioNumerics (version 7.1; Applied Maths, Sint-Martens-Latem, Belgium). Spectra were initially pre-processed using the default program settings (Baseline Subtraction). For cluster analysis, spectra were compared pairwise using the Pearson correlation coefficient. Dendrograms were generated using the UPGMA algorithm. MDS analysis was performed as previously described to visualize the similarity between spectra (Goldstein et al., 2013). Jackknife analysis was performed as described before (Giebel et al., 2008) using average similarities to quantify rates of correct classification with regards to MALDI-TOF MS (**Table 2**).

**Table 2:** Assignment of isolates to sample source by using Jackknife Average Similarities analysis and MALDI-TOF spectra or BOX-A1R PCR fingerprints.

Assigned group	% of *D. aquaticus* isolates in assigned group^+^	
**Jackknife and MALDI-TOF MS comparisons**	**River^∗^**	**Lake^#^**
River^∗^	50.00	19.44
Lake^#^	50.00	80.56

**Jackknife and BOX-A1R PCR fingerprints**	**River^∗^**	**Lake^#^**
River^∗^	100.00	83.3
Lake^#^	0.00	16.7

*^+^values in bold indicate percentages of isolates correctly assigned to sources groups; ^∗^Fox River; ^#^Lake Winnebago.*

## Results and Discussion

### MALDI-TOF Affords Rapid Isolate-Level Characterization

**FIGURE 2:**
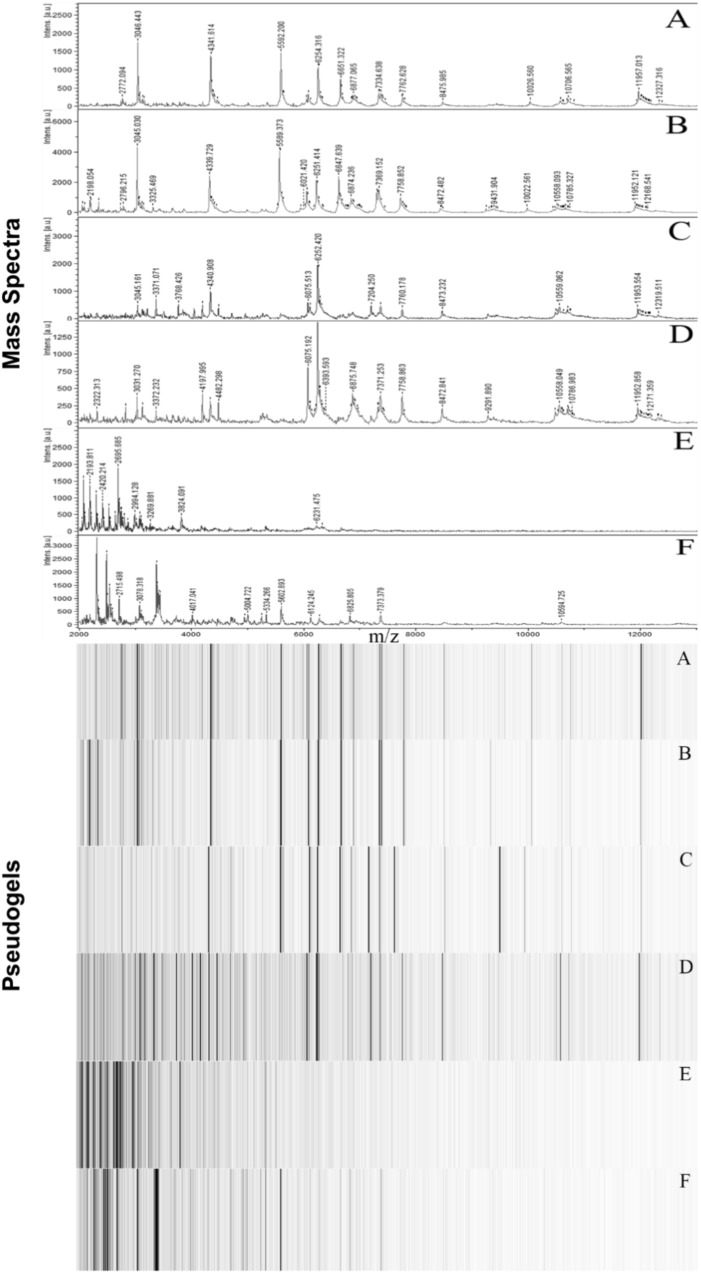
Representative MALDI-TOF mass spectra and corresponding pseudogel views of Fox River and Lake Winnebago *Deinococcus* isolates. **(A)** *D. aquaticus* isolate P21; **(B)** *D. aquaticus* isolate P22; **(C)** *D. aquaticus* isolate P76; **(D)** *D. aquaticus* isolate P81; **(E)** *D. misasensis* isolate P49; and **(F)** *D. gobiensis* (control of the study).

Matrix-assisted Laser Desorption Ionization-Time of Flight Mass Spectroscopy analysis resulted in unique spectra for each *D. aquaticus* isolate originating from different biofilm communities indigenous to diverse substrates (concrete, leaf tissue, metal, and wood) in the Fox River system of Wisconsin (**Figure 1**). We examined a mass range of m/z 2,000–20,000, although we report data only for the m/z range of 2,000–13,000 since no peaks were detected outside of this range (**Figure 2**). Many studies utilize a narrower range when characterizing microbial isolates via MALDI (Dieckmann et al., 2008; Ferreira et al., 2011; Emami et al., 2012), but we employed a broader mass range that contains peaks that appear characteristic at species and isolate level. **Figure 2F** which corresponds to the spectrum of a *D. gobiensis* strain has several peaks (m/z 2315, 2589, 2715, 3078, and 3378) within an m/z range of 2,000–4,000 that are unique compared to *D. aquaticus* isolates (**Figures 2A–D**). Also, isolate P49 (**Figure 2E**), which was originally thought to be *D. aquaticus* but later was proven to be *D. misasensis* (Asker et al., 2008), was readily differentiated within the same m/z range (m/z 2193, 2420, 2695, 2994, and 3269) from the rest of the isolates (**Figures 2A–D**). Thus, aside from the aforementioned discrimination at isolate level, we also observed discrimination at the species level (*D. aquaticus*, *D. gobiensis*, and *D. misasensis*). The mass spectra of all bacterial isolates along with their corresponding pseudogels and analysis of MS peak classes using matrix mining (heat maps) can be found in the **Supplementary Data Sheet 1**.

### Comparison of MALDI-TOF to More Established Methods

**FIGURE 3:**
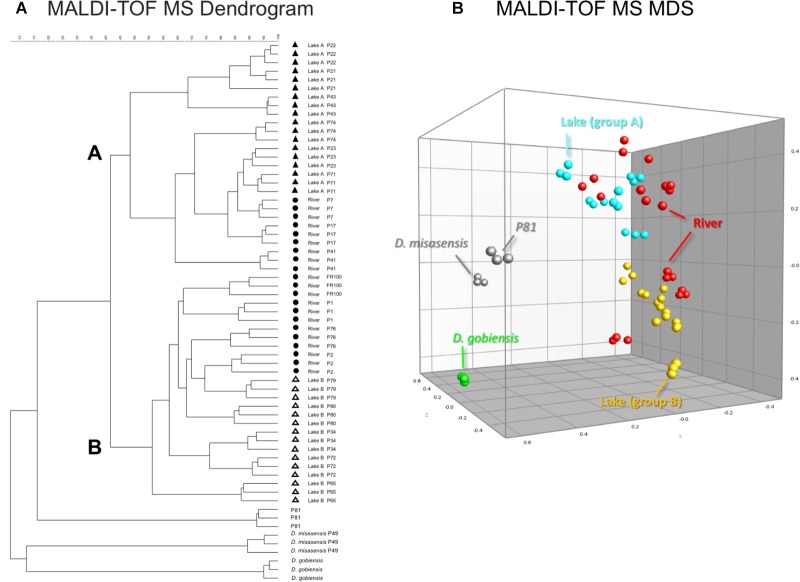
MALDI-TOF MS spectra-based dendrogram and multidimensional scaling (MDS) representation of *Deinococcus* isolates of the study. **(A)** Dendrogram was constructed using the UPGMA algorithm. Two clusters A and B were identified associated with lake isolates. The river isolates clustered with either clusters A or B. **(B)** MDS representation of the MS data. Lake isolates clustering with group A and B are light blue and yellow, respectively, river isolates are red, *D. gobiensis* (control) is green, *D. misasensis* and isolate P81 (outlier) are gray.

Differences observed in the representative mass spectra shown in **Figure 2** were reflected in the cluster analysis of the spectra of all 19 *Deinococcus* isolates studied here (**Figure 3**). Spectra of isolates clearly separated at species level, since *D. aquaticus* isolates separately from *D. misasensis* and *D. gobiensis*. We did not observe distinct clusters among the *D. aquaticus* isolates based on the substrate or location. However, two major groups were identified, denoted as A and B, with >40% similarity to each other. These two groups are composed of isolates from both the Fox River and Lake Winnebago. Therefore, we hypothesize that groups A and B result from dominant co-occurring populations (**Figure 1**), which can be differentiated via MALDI-TOF analysis. Additionally, the MALDI-TOF spectra illustrate population stability with high similarity between a 2010 and 2013 isolate (FR100 and P1) (**Figure 3A**). The detection of a single outlier, P81, in the MALDI-TOF analysis provides evidence for greater phenotypic diversity within *D. aquaticus*. However, due to the level of sampling from these environments, we do not know if the spectra for P81 may represent an additional grouping for this species. The microdiversity detected within each group illustrates that MALDI-TOF provides a fine level of taxonomic resolution when identifying members of the same species. From the ecological and evolutionary perspective, it is noteworthy that this microdiversity was preserved despite the rigors of laboratory sub-culturing and continual passage of these environmental isolates.

**FIGURE 4:**
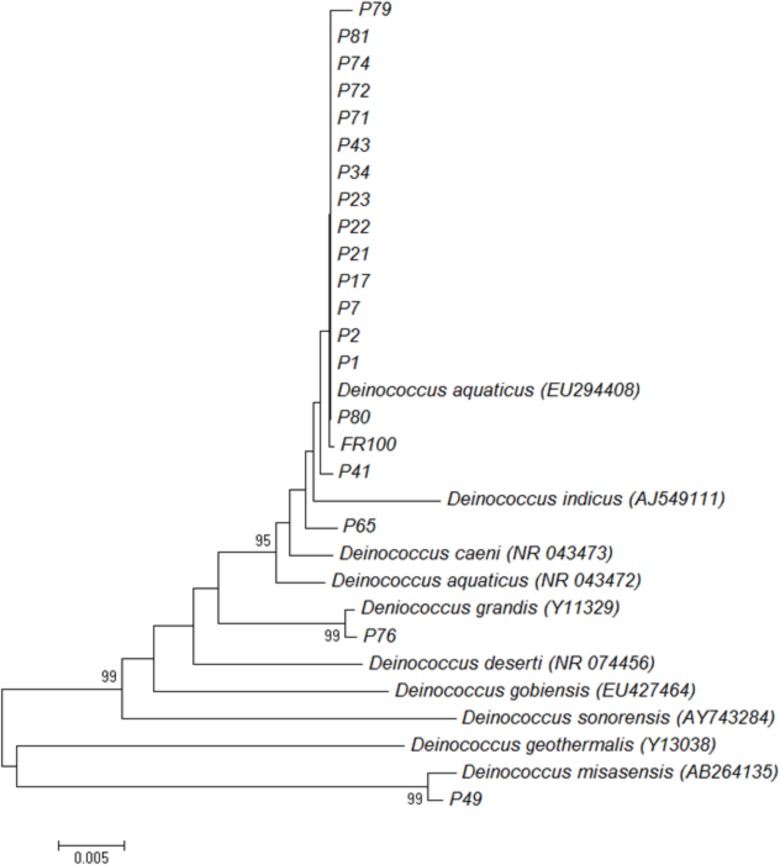
Neighbor-joining phylogenetic tree based on 16S rRNA gene sequences showing relationships between isolates and representative *Deinococcus* species. The numbers on the nodes illustrate percentages of bootstrap support based on 100 replicates. Only values above 75% are given.

We also performed 16S rRNA gene sequence analysis and compared it to the MALDI-TOF MS data. The 16S rRNA-based dendrogram (**Figure 4**) revealed one major clade corresponding to all *D. aquaticus* isolates regardless of the isolation surface substrate or the body of water that was sampled. *D. gobiensis* and isolate P49 (*D. misasensis*, **Table 1**) clustered separately from the rest of the isolates and this observation was consistent with the MALDI-TOF spectrum (**Figure 2E**) and the dendrogram (**Figure 3**), supporting the sensitivity of this method for species-level distinction. Interestingly, P76 isolate uniquely originated from a natural surface substrate in Fox River (leaf, **Table 1**), clustered with *D. grandis* in the 16S rRNA-based phylogenetic tree (**Figure 4**), whereas in the MALDI-TOF-based dendrogram, it clustered together with group B (**Figure 3**) and in the Box-A1R, it clustered with *D. aquaticus* isolate P17. Substrate specific-clustering (concrete, leaf tissue, metal, and wood) was not observed with either 16S rRNA or MALDI-TOF approaches. Of note, in our previous studies where we compared MALDI-TOF MS and 18S rRNA gene sequences of microalgae cultures, we also observed that the differences in the two methods are reflective of the facts that 18S rRNA data are based only on a single gene sequence, while MALDI data contain proteome-level information (Barbano et al., 2015). Further work is warranted to determine the precise potential of these two techniques in their ability to characterize bacterial isolates of the same species that are grown on different substrates.

**FIGURE 5:**
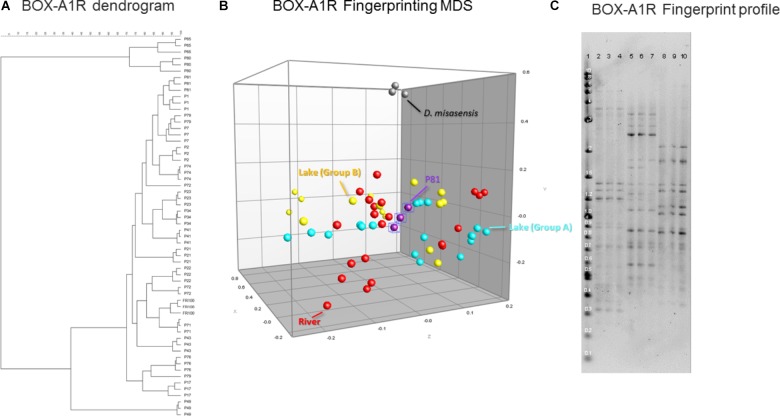
Similarity-based dendrograms representing study isolates by BOX-A1R PCR fingerprints **(A)** and multidimensional scaling (MDS) representation **(B)**. Lake isolates clustering with group A and B are light blue and yellow, respectively, river isolates are red, *D. misasensis* is gray and isolate P81 (outlier) are purple. Fingerprint patterns of representative *D. aquaticus* isolates, obtained with the BOX-A1R primer. **(C)** Lane: 1, 2-Log DNA Ladder (Kb); 2, 3, and 4, isolate P17; 5, 6, and 7, isolate P21; 8, 9, and 10, isolate P22.

Estimates of genomic diversity as assessed by repetitive DNA sequences (BOX-A1R fingerprinting) to differentiate *Deinococcus* isolates did not facilitate discrimination of the isolates either based on the surface substrate (e.g., concrete, leaf tissue, metal, and wood), isolation location, or the body of water (e.g., lake vs. river). In general, there was greater diversity detected utilizing this genomic method, segregating the collection into several groups (**Figure 5A**). This further supports the thesis that the BOX-A1R fingerprinting technique has nuances that limit its robustness for assessing strain differentiation.

River-lake ecosystems are highly heterogeneous in terms of both geomorphology and hydrodynamics. While we presume that the water flow shapes the physical architecture of the biofilms where these isolates where extracted, it is impossible to know what drives the community assembly and biodiversity of these biofilms in the heterogeneous flow landscapes of the Fox River – Lake Winnebago system of Wisconsin. We postulate that a number of broad range environmental factors such as water temperature, pH, nutrients, dissolved organic carbon, and environmental pollutants are the potential drivers of biofilm community composition and diversity that may be reflected in our data.

In our present study, we show that MALDI-TOF MS analysis can be used as an efficient, inexpensive, and reliable tool to identify and characterize *D. aquaticus* isolates that originate from specific niches, such as a freshwater system yielding distinct banding profiles with fragments ranging from 0.3–5 kb (**Figure 5C**). The relatively high cost and time-intensive approaches of BOX-A1R fingerprinting and of 16S rRNA gene sequencing failed to differentiate *D. aquaticus* isolates from different surfaces, body of water, or isolation location. These findings are corroborated by our MDS representation of MALDI-TOF spectra (**Figure 3B**), in which it is readily apparent that that there are two different, yet co-occurring groups of isolates (group A and B) originating from lake water and river water. The same MDS analysis based on BOX-A1R fingerprinting did not differentiate the *D. aquaticus* isolates from the two different bodies of water (**Figure 5B**) or different surface substrates (data not shown). A higher rate of correct classification with regard to lake water isolates was observed with MALDI-TOF MS (80.56%) compared to BOX-A1R fingerprints (16.7%) (**Table 2**). The low percentage for the river isolates (50%) is consistent with the MALDI-TOF derived dendrogram and MDS (**Figures 3A,B**) in which river isolates cluster either with lake groups A or B. The correct classification into isolation habitat based upon the MALDI-TOF analysis, suggests that lake isolates may show more plasticity.

Clearly, the respective biological targets of the MALDI-TOF MS and BOX-A1R fingerprinting techniques are fundamentally different. One addresses genotype, while the other addresses phenotype. It is reasonable to assume that the repetitive element distribution can generate closely related patterns of genomic diversity, whereas the phenotypic profiles are reflective of physiological differences that may impact ecological success and be habitat-specific. Also, in our previous studies when we compared MALDI-TOF MS data with BOX PCR fingerprinting data in environmental isolates of *Escherichia coli* in dendrograms constructed using the UPGMA we found different clusters between the two methods (Siegrist et al., 2007).

Several prior studies have already shown that MALDI-TOF MS is a robust and efficient tool to differentiate and identify bacterial isolates from different ecosystems. For example, researchers have successfully used MALDI-TOF MS to differentiate bacterial species of the *Rhizobiaceae* family, divided them in three genera, *Rhizobium*, *Ensifer*, and *Shinella*, and established their pathogenic, saprophytic and symbiotic interactions with plants (Ferreira et al., 2011). In another study MALDI-TOF MS was used to assess the cultivable diversity of environmental prokaryotes. They managed to acquire strain-specific spectra and grouped halophilic and aerobic prokaryotes into distinct clusters associated with different taxa (Munoz et al., 2011). Furthermore, MALDI-TOF MS has been used to detect contamination of the natural environment by identifying bacterial species metabolizing biphenyl from contaminated horseradish rhizosphere soil (Uhlik et al., 2011) and sewage sludge (Ruelle et al., 2004).

In summary, we have shown that the use of MALDI-TOF MS technology is sufficient not only to discriminate between *Deinococcus* species but also to differentiate between *Deinococcus* isolates, highlighting the microdiversity present in closely related strains. The technique appears to provide advantages over existing genomic techniques in terms of resolving power and ease of use and recommends itself as a tool in environmental studies of this type.

## Ethics Statement

All protocols used in this study were evaluated and approved by the Arizona State University, University of Wisconsin Oshkosh, and Glendale Community College.

## Author Contributions

JT and SM-S designed the study, performed experiments, ran statistics, analyzed data, and contributed to the manuscript writing. CA, SS-N, and MW, performed experiments, ran statistics, and analyzed data. GN ran statistics, analyzed and synthesized the data, and contributed to the manuscript writing. AG assisted in acquisition of mass spectra and BioNumerics analysis. TS provided oversight to the entire project, involved in study design, data analysis, integration, and writing of the manuscript.

## Conflict of Interest Statement

The authors declare that the research was conducted in the absence of any commercial or financial relationships that could be construed as a potential conflict of interest.

## Footnotes

1 http://www.bacterio.net/deinococcus.html

## Abbreviations

BOX-A1R fingerprinting: repetitive sequence-based polymerase chain reaction fingerprints obtained by using primer BOX-A1R (5′-CTACGGCAAGGCGACGCTGACG-3′)
CFU: colony forming units
*D. aquaticus*: *Deinococcus aquaticus*
*D. gobiensis*: *Deinococcus gobiensis*
*D. misasensis*: *Deinococcus misasensis*
ddH_2_O: double distilled water
MALDI-TOF MS: Matrix-assisted Laser Desorption Ionization-Time of Flight Mass Spectroscopy
MDS: multidimensional scaling
RT: room temperature
UPGMA: unweighted pair group method with arithmetic mean
16S rRNA gene: 16S ribosomal RNA gene.
